# Edinburgh Surgical Hospital

**Published:** 1831-05

**Authors:** 


### EDINBURGH SURGICAL HOSPITAL.

*Reunion of Fractured Bones.* [From Mr. Syme's Report; *Edinburgh Med. Journal.*]

It is daily observed in treating fractures of long bones, such as the tibia and femur, that, notwithstanding the most careful and effectual means are employed to retain the corresponding surfaces in situ, they remain moveable for many days, and, indeed, generally for the best part of three weeks; during the whole of which period the crepitation heard or felt by moving the limb is as distinct as immediately after the injury has occurred. The mobility usually ceases very suddenly, and the limb all at once regains such a degree of firmness as to sustain its own weight, or resist any other equivalent force tending to bend it; but if subjected to more considerable violence at this time, it gives way again at the part originally fractured. When such fractures are dissected within the first two or three weeks of their existence, the ends of the bones are found quite separate and unconnected by any intermediate substance. These facts are quite opposed to the idea, that the uniting process consists entirely in the effusion and ossification of a substance proceeding from the surfaces of the bones, in which case the mobility should diminish gradually, and flexibility continue long after perfect mobility had ceased, before the establishment of perfect rigidity.

Case I. Catherine Adams, æt. fifty-two, was admitted on the 12th of January, soon after sustaining a fracture of the right thigh-bone, in its lower third, by falling on her side. Pasteboard splints were applied to keep the limb steady, and then, by means of the long splint of Desault, extension was effected, so as to prevent retraction of the broken surfaces, which were very oblique. Every thing appeared to be doing well until the 23d of January, when she had a long and severe rigor, and afterwards complained of general uneasiness, with the other usual symptoms of fever. On the following day, her tongue was brown, and hard; her pulse frequent, but weak; and her appearance, upon the whole, extremely unpromising. Thinking that she would not bear bleeding, I desired that she should have her bowels freely opened by injections, and afterwards take small doses of an antimonial solution. On the 25th, she complained of her throat being very sore, and her respiration was performed with the peculiar sound which indicates œdema of the glottis. Though this symptom was very distinctly marked, it did not seem to warrant tracheotomy, as there was no indication of any severe degree of obstruction in the breathing, and the patient appeared to be sinking independently of this local disease. I, therefore, directed blisters to be applied to the throat, and stimulants to be given frequently. She died next day.

On dissection, the fracture was found to extend obliquely from near the middle of the bone down to the external condyle. The muscular fibres and cellular substance in the neighbourhood of the injury were altered in colour, as well as consistence, by the effusion of gelatinous matter into their texture. A kind of bag, or capsule, was thus formed, embracing the whole extent of broken surfaces, and containing two or three ounces of fluid blood. The parietes composing it were in some parts connected to the very edge of the bone, but in others they became adherent to it at a distance of an inch or more from the extremity, leaving a space to this extent uncovered, and apparently denuded of periosteum. When carefully examined, this exposed portion was ascertained to be covered by a thin layer of gelatinous substance, which did not possess the toughness or other characters of a membrane; and the respective surfaces of the bone had a covering of the same kind. The medullary membrane was very vascular, and more distended than usual.

In examining the structure of this bag, I endeavoured to ascertain which of the natural tissues entered into its formation, and in what parts of it, if any, ossification had commenced. On tracing the periosteum from the sound bone, I found, that where the bag adhered, that membrane became thick, and evidently continuous with its walls. It seemed probable that, where the membrane had been stripped off the bone, as already mentioned, it might assist to form, in some small part, the sac in question; the great extent of which, however, was evidently constituted by the neighbouring tissues, whatever they happened to be, muscle, tendon, fat, or cellular substance, all being reduced to the same appearance internally, by vascularity of the surface, and the same consistence, by the interstitial effusion of organizable matter.

On introducing my finger into the bag, so as to feel if there were any indications of ossification, I perceived some small grains or specks of bone, which, when minutely examined, presented a stellated appearance, and were ascertained to lie in the substance of the capsular membrane. When examined, in the same way, near its connexion with the bone, it was found to contain much larger masses possessing osseous firmness; in order to ascertain the precise seat and origin of which, I carefully dissected the membrane where they existed, and then found that they lay completely imbedded within it, having a covering from it on both sides: also that they did not adhere to the bone, being separated from it by a thin layer of the membrane, so as to admit of a slight degree of motion; but at these parts the shaft itself had begun to shoot out a growth of new bone.

Case II. Mary Donaldson, a poor emaciated old woman, seventy years of age, was admitted on the 27th of September, on account of a compound fracture of the left leg close to the ankle. Both the tibia and fibula were shattered into many fragments, and there was a wound over the latter bone extending down to it. Pasteboard splints were applied, the limb being laid on its outer side with the knee bent; but the patient proved so unmanageable and undocile in favoring the maintenance of steadiness in this position, that I was obliged to have recourse to Macintyre's inclined plane, which answered the purpose perfectly. She made no complaint afterwards, and all her functions were performed in a natural manner. For nearly three weeks crepitus could be distinctly felt when the limb was moved; but then the bones united, the wound healed, and on the 25th of October the cure seemed to be complete. The splints were then removed, and a simple roller applied. On the 5th of November, she was dismissed with the limb perfectly straight.

About ten days afterwards, I was much surprised to learn that she had died in consequence of some internal disease, and having procured permission to dissect the limb that had been fractured, obtained possession of the bones for their more careful examination. When divested of their muscular coverings, they presented an appearance hardly differing from that naturally belonging to them. All the pieces into which they had been broken were firmly united to each other and to their shafts, and were covered with a periosteum of usual consistence. On closer examination, the interstices between these portions were found to be occupied by a soft bloody gelatinous substance, to ascertain the precise extent of which the preparation was macerated. When all the interstitial matter had been thus separated, it was seen that the united fragments of the tibia, which were thirteen in number, constituted merely a skeleton, so to speak, of the cylinder, and that the central cavity remained entirely vacant. On examining the internal surface of this imperfect shell, it was evident that an ossific process had been going on over the whole of it; and I have no doubt that, if the patient had lived some months longer, the bones would have become completely solid. The fibula presented similar appearances, though on a smaller scale, and the process of reunion was more nearly perfected. There is in my possession the preparation of a thigh-bone which was fractured through the neck and trochanters, and was treated by my friend Mr. George White. The patient died two months after the accident, from some other cause. It now appears, the bone having been macerated, that all the broken portions are firmly united together at the edges, but that all their internal surfaces remain perfectly distinct and separate. The appearance, in short, is very nearly the same, and, I believe, would also have terminated in compact ossification, if the necessary time had been afforded.

I will take the first opportunity of returning to this subject, and endeavour to elucidate it further. In the meantime, it may not be improper to remark, that the delay which would appear to occur before the broken surfaces of the bones are united, affords no reason or excuse for the practice that is sometimes recommended, of deferring the use of any means to keep the limb in proper position, until ten or twelve days have elapsed after the accident. The first step in the process of reparation seems to be thickening of the surface presented by the surrounding tissues, from effusion into them; and as this, we have reason to believe, is commenced immediately, without a day's delay, if the surgeon defers setting the bones, he will not only lose the assistance thus afforded in preventing their displacement, but also run the risk of allowing the broken extremities to become so far fixed in the distorted situations into which they have been driven by the contraction of the muscles, or the weight of the limb, as to prevent their perfect adjustment by any force which he has it in his power to employ.

